# Experience of patent ductus arteriosus ligation during extracorporeal membrane oxygenation treatment in newborns with severe respiratory failure due to persistent pulmonary hypertension: a single-center retrospective study

**DOI:** 10.1186/s13052-024-01821-8

**Published:** 2024-11-22

**Authors:** Qi-Liang Zhang, Yi-Nan Liu, Ya-Ting Zeng, Yi-Rong Zheng, Qiang Chen

**Affiliations:** Department of Cardiac Surgery, Fujian Children’s Hospital (Fujian Branch of Shanghai Children’s Medical Center), College of Clinical Medicine for Obstetrics & Gynecology and Pediatrics, Fujian Medical University, Fuzhou, China

**Keywords:** Newborns, ECMO, PDA ligation, Early results

## Abstract

**Background:**

The aim of this study is to summarize our center’s experience with patent ductus arteriosus (PDA) ligation during extracorporeal membrane oxygenation (ECMO) treatment in newborns with severe respiratory failure due to persistent pulmonary hypertension of the newborn (PPHN).

**Methods:**

We retrospectively collected and analyzed clinical data from five newborns with severe respiratory failure due to PPHN who underwent PDA ligation during ECMO treatment at our hospital between January 2021 and August 2023.

**Results:**

All five patients had large PDAs, measuring 10 mm, 6 mm, 6 mm, 7 mm, and 6 mm, respectively. Significant left-to-right shunting through the PDA was observed after 29 h, 14 h, 3 h, 7 h, and 5 h of ECMO treatment, respectively, at which point successful PDA ligation was performed. The surgical durations were 52 min, 45 min, 55 min, 50 min, and 40 min, respectively. Post-ligation, blood lactate levels significantly decreased compared to preoperative values. Four patients were successfully weaned off ECMO, with ECMO support durations of 64 h, 92 h, 70 h, and 87 h, respectively. After ECMO removal, mechanical ventilation was discontinued after 5.2 days, 7.2 days, 9.5 days, and 5.5 days, respectively. None of the four surviving patients experienced complications such as residual shunting, bleeding, chylothorax, neurologic injury, pneumothorax, poor wound healing, or sepsis.

**Conclusion:**

During ECMO treatment for PPHN in newborns with large PDAs, the direction of blood flow through the PDA should be closely monitored. PDA ligation is a feasible and reasonable intervention when pulmonary artery pressure decreases and left-to-right shunting through the PDA becomes evident.

## Introduction

Persistent pulmonary hypertension of the newborn (PPHN) is characterized by a sustained increase in pulmonary vascular resistance after birth, leading to right-to-left shunting of blood at the atrial and/or ductal levels, resulting in severe hypoxemia and a high mortality rate [1, 2]. The conventional treatment for PPHN includes mechanical ventilation support, maintenance of normal systemic circulatory pressure, administration of vasodilators, and inhaled nitric oxide. However, in cases of severe PPHN that do not respond to standard treatments and meet the criteria for extracorporeal membrane oxygenation (ECMO) application, ECMO support should be considered [3–6]. Newborns with PPHN often experience severe hypoxemia, acidosis, and pulmonary hypertension, which can lead to heart failure and circulatory instability. Venoarterial (V-A) ECMO provides both respiratory and cardiac support, making it the preferred mode of treatment for patients with PPHN [7, 8]. 

Patent ductus arteriosus (PDA) is a common vascular anomaly in newborns, where a connection persists between the descending aorta and the pulmonary artery [9, 10]. The presence of a PDA can lead to blood shunting, particularly in cases of large PDAs with significant left-to-right shunting, which may result in pulmonary congestion, pulmonary hemorrhage, reduced systemic blood volume, inadequate systemic perfusion, elevated blood lactate levels, necrotizing enterocolitis, and other related complications [11, 12]. These patients often require surgical closure of the PDA. Newborns with severe respiratory failure due to PPHN frequently have a concomitant PDA, which may present as a right-to-left or bidirectional shunt. When ECMO treatment is initiated, pulmonary artery pressure may decrease, resulting in left-to-right shunting through the PDA. In cases where the shunt volume through the PDA is large, it can lead to hemodynamic instability during ECMO treatment, necessitating surgical closure of the PDA. However, there is limited literature reporting on the experience of PDA ligation during ECMO treatment, with most accounts consisting of case reports. This study summarizes the experience of PDA ligation during ECMO treatment in five cases of severe respiratory failure due to PPHN at our center and reports early clinical outcomes.

## Methods and materials

Clinical data were collected from five newborns with severe respiratory failure due to PPHN who underwent PDA ligation during ECMO treatment at our hospital between January 2021 and August 2023. This study was approved by the hospital’s ethics committee, and informed consent was obtained from the patients’ families.

### ECMO cannulation technique

All patients underwent bedside cannulation for ECMO in our cardiac intensive care unit. Right carotid artery and right internal jugular vein cannulation were performed in all cases. The arterial cannulas (Medtronic, USA) were all 8 French (F), and the venous cannulas (Medtronic, USA) were all 10 F.

### PDA ligation technique

**Fig. 1:**
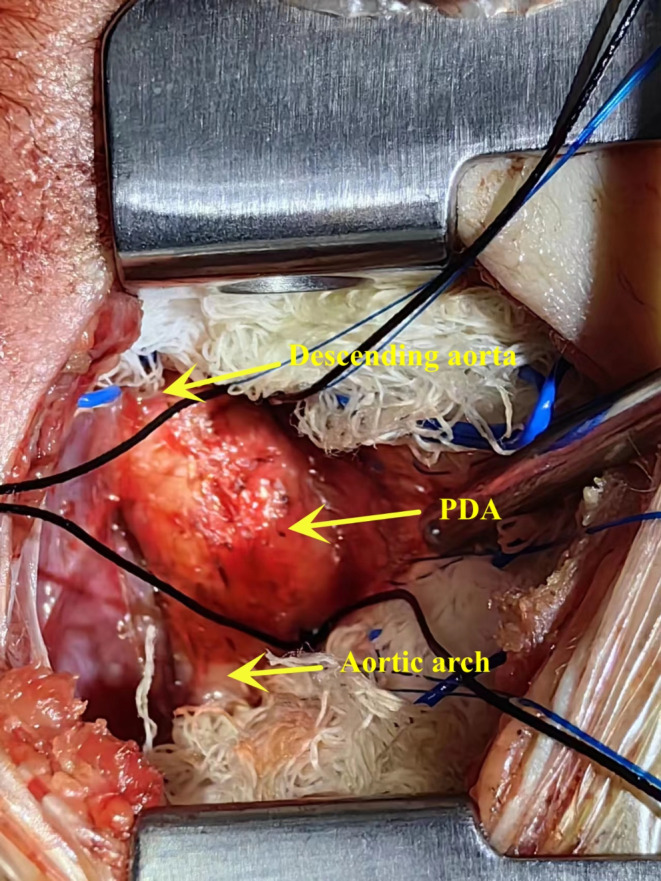
The exposure of PDA

After anesthesia, patients were carefully positioned in the right lateral decubitus position. Special attention was given to maintaining ECMO flow, and the ECMO circuit was securely fastened. A 2 cm incision was made below the outer edge of the left scapula, extending 3–4 cm in an arc toward the spine. The skin, subcutaneous tissue, and muscles were sequentially dissected, and the chest was entered through the fourth intercostal space. Respirator settings, including positive end-expiratory pressure (PEEP) and tidal volume, were reduced to allow for lung collapse. The mediastinal pleura was incised longitudinally from the origin of the descending aorta near the left pulmonary artery to the pulmonary hilum, and it was then retracted. The ductus arteriosus was identified near the origin of the descending aorta, close to the left pulmonary artery, and was carefully dissected free at its upper and lower poles, revealing a ductus arteriosus approximately 1 cm wide and 1 cm long (Fig. 1). After separating the space between the upper and lower margins of the PDA with angled forceps, two 1 − 0 sutures were placed around the PDA, one above and one below. The PDA was ligated, when sodium nitroprusside was applied to reduce the systolic blood pressure to 40-50mmHg. After confirming that there was no bleeding, the mediastinal pleura was sutured to achieve hemostasis, and a chest tube was left in place. The chest was closed layer by layer, including the muscle, subcutaneous tissue, and skin. Patients were then carefully repositioned, and the ECMO circuit was re-secured.

## Results

The gestational ages of the five newborns in this study were 39.7 weeks, 37.7 weeks, 39.6 weeks, 38.4 weeks, and 35.3 weeks, respectively, and their birth weights were 3280 g, 2740 g, 2375 g, 3805 g, and 2700 g, respectively. All presented with PPHN shortly after birth, leading to severe hypoxemia and heart failure. ECMO was initiated at 1 day, 1 day, 3 days, 2 days, and 5 days, respectively. All five cases were complicated by large PDAs, with sizes of 10 mm, 6 mm, 6 mm, 7 mm, and 6 mm, respectively. The pulmonary artery pressures before ECMO were 65 mmHg, 78 mmHg, 66 mmHg, 70 mmHg, and 69 mmHg, respectively, and lactate levels were 8 mmol/L, significantly elevated, 12 mmol/L, 9 mmol/L, and 13 mmol/L, respectively. The direction of blood flow through the PDA was a right-to-left shunt, and no pulmonary hemorrhage was observed (Table 1).

**Table 1:** Clinical data of patient during ECMO period

	Gestational age (week)	Birth weight (g)	Age (d)	Pulmonary artery pressure (mmHg)	Size of PDA (mm)	Pneum- orrhagia before PDA ligation	Blood flow direction of PDA	Lactic acid (mmol/L)
Patient 1	39.7	3280	1	65	10	No	right -to- left shunt	8
Patient 2	37.7	2740	1	78	6	No	right -to- left shunt	High
Patient 3	39.6	2375	3	66	6	No	right -to- left shunt	12
Patient 4	38.4	3805	2	70	7	No	right -to- left shunt	9
Patient 5	35.3	2700	5	69	6	No	right -to- left shunt	13

After ECMO support, pulmonary artery pressure decreased in all patients, and the left-to-right shunt through the PDA gradually increased, while lactic acid levels did not decrease. During this period, three patients developed symptoms of pulmonary hemorrhage. Significant left-to-right shunting through the PDA was observed in all patients after 29 h, 14 h, 3 h, 7 h, and 5 h of ECMO treatment, respectively, prompting the decision to perform PDA ligation. The surgical durations were 52 min, 45 min, 55 min, 50 min, and 40 min, respectively. Before PDA ligation, the patients’ platelet counts were 67 × 10^9^/L, 82 × 10^9^/L, 178 × 10^9^/L, 129 × 10^9^/L, and 328 × 10^9^/L, respectively. The activated clotting times (ACT) were 198 s, 200 s, 186 s, 190 s, and 210 s, respectively. Intraoperative bleeding volumes were 10 ml, 10 ml, 2 ml, 5 ml, and 5 ml, respectively. After PDA ligation, all patients showed a significant decrease in blood lactate levels compared to preoperative values. Four patients were successfully weaned off ECMO, with ECMO support durations of 64 h, 92 h, 70 h, and 87 h, respectively. After ECMO removal, mechanical ventilation was discontinued after 5.2 days, 7.2 days, 9.5 days, and 5.5 days, respectively. One patient experienced intracranial hemorrhage on the second day after PDA ligation, and the family decided to discontinue further treatment, resulting in the cessation of care (Table 2).

**Table 2:** Clinical data of patient in perioperative period of patent ductus arteriosus ligation

	Pulmonary artery pressure before PDA ligation (mmHg)	Pneum- orrhagia before PDA ligation	Blood flow direction of PDA	Platelet before PDA ligation (/L)	ACT before PDA ligation (s)	Operation time of PDA ligation (min)	Amount of bleeding (ml)	Lactic acid before PDA ligation (mmol/L)	Lactic acid in 1 h after PDA ligation (mmol/L)	Lactic acid in 3 h after PDA ligation (mmol/L)	Pneum- Orrhagia after PDA ligation	Time of ECMO treatment (h)
Patient 1	55	Yes	left-to- right shunt	67*10^9	198	52	10	10.4	6.6	5.1	Reduction	64
Patient 2	65	Yes	left-to- right shunt	82*10^9	200	45	10	High	High	29	Reduction	Abandon treatment
Patient 3	53	Yes	left-to- right shunt	178*10^9	186	55	2	15	11.9	10.5	Reduction	92
Patient 4	60	No	left-to- right shunt	129*10^9	190	50	5	11.1	4.5	2.7	-	70
Patient 5	59	No	left-to- right shunt	328*10^9	210	40	5	15	7.8	4.8	-	87

None of the four surviving patients experienced complications such as residual shunt, major bleeding, chylothorax, neurological injury, pneumothorax, poor wound healing, or sepsis.

## Discussion

Pulmonary artery hypertension occurs in 2–5 of every 1,000 live births [13]. Acute pulmonary hypertension, which leads to hypoxemic respiratory failure during the transitional period, is characterized by a failure of the normal postnatal decline in pulmonary vascular resistance. The hypoxemia is related to impaired pulmonary blood flow and/or right ventricular dysfunction secondary to elevated pulmonary artery pressure and increased right ventricular afterload [14]. Consequently, severe PPHN in newborns often leads to profound hypoxemia, ultimately resulting in life-threatening circulatory and respiratory failure, with a mortality rate of approximately 50%[15, 16]. If respiratory failure persists despite standard treatment, ECMO therapy becomes necessary [17]. 

Cardiac dysfunction is more pronounced in neonates due to an underdeveloped contractile system with decreased compliance, reduced adaptability to changes in afterload, and an increased risk of diastolic dysfunction [18]. In the context of severe acute pulmonary hypertension, where there is significant right ventricular dysfunction and/or extremely low cardiac output, a continuous right-to-left shunt through the PDA can play a supportive role by reducing right ventricular pressure. The PDA should remain open during this time [19]. As treatment progresses and pulmonary vascular resistance and pulmonary artery pressure decrease, the PDA may develop a left-to-right shunt. Numerous studies have shown that when the PDA exhibits hemodynamically significant left-to-right shunting, closure of the PDA is necessary, as it can lead to complications such as pulmonary hemorrhage, renal failure, necrotizing enterocolitis, and even neonatal mortality [20, 21]. Hemodynamically significant PDA (HsPDA) is typically defined as a PDA with significant left-to-right shunting through the ductus arteriosus, confirmed by echocardiography and clinical evidence of systemic hypoperfusion and pulmonary overcirculation [22, 23]. For newborns with large PDAs, during ECMO treatment, when pulmonary artery pressure decreases, a large PDA can result in significant left-to-right shunting, leading to pulmonary congestion, hemorrhage, and reduced systemic circulation, among other adverse effects. Therefore, surgical closure of the PDA should be considered in newborns with severe PPHN and concomitant large PDAs during ECMO support. However, performing PDA ligation during ECMO carries a high risk. This study summarizes our clinical experience with performing PDA ligation during ECMO therapy in newborns.

Since PPHN in newborns is often self-limiting, pulmonary artery pressure decreases as the infant ages. Therefore, when ECMO-assisted therapy reduces pulmonary artery pressure to the point where left-to-right shunting occurs through the PDA, surgical closure of the PDA should be considered. If the PDA is large at this point, it can result in significant left-to-right shunting, leading to pulmonary congestion, pulmonary hemorrhage, and adverse effects on the already compromised lung function and the management of pneumonia, which can hinder successful ECMO removal [24, 25]. A significant reduction in systemic circulation can also lead to inadequate systemic perfusion, tissue ischemia, hypoxia, and complications such as elevated lactate levels and necrotizing enterocolitis [26]. Therefore, for newborns with severe respiratory failure and large PDAs undergoing ECMO-assisted therapy, closure of the PDA should be considered when pulmonary artery pressure decreases to the point where left-to-right shunting begins [24]. If significant pulmonary congestion, pulmonary hemorrhage, or elevated lactate levels occur, PDA ligation should be performed promptly. In the cases discussed in this study, all five patients had PDAs measuring 6 mm to 10 mm, which were close to or even exceeded the diameter of the aorta. Before PDA ligation, all five patients exhibited continuously rising lactate levels, and three experienced pulmonary hemorrhages. After PDA ligation, lactate levels significantly decreased, and pulmonary hemorrhages gradually resolved.

Due to the invasive nature of PDA ligation surgery and the need for heparin anticoagulation during ECMO therapy, the risk of intraoperative bleeding is relatively high [27]. To prevent bleeding, careful surgical techniques, avoidance of vascular and unnecessary tissue injury, and meticulous hemostasis are essential. We followed the approach recommended by Wang G, et al., [28] which involves maintaining the ACT within the range of 180–220 s, ensuring platelet counts above 60 × 10⁹/L, and using sodium nitroprusside to control the systolic blood pressure at 40–50 mmHg during PDA ligation. None of the five patients in our study experienced severe bleeding complications, with intraoperative bleeding volumes not exceeding 10 ml.

To improve the success rate of the surgery and minimize complications, we summarized the following key points from our experience: optimal exposure and a clear surgical field were crucial for the procedure’s success. Since ECMO support was available for cardiopulmonary function, we temporarily suspended mechanical ventilation during chest entry, retaining PEEP, and resumed ventilation only after completing the PDA ligation. This approach prevented respiratory movements from obstructing the surgical field, significantly improving visibility without causing circulatory changes. It is important to note that this brief pause in ventilation did not lead to lung perfusion issues, as the surgery duration was short. Newborns often had significant tissue edema due to their young age, heart failure, and ECMO support, making the aorta and PDA tissue very thin and susceptible to damage. Therefore, it was essential to perform the surgery meticulously and gently. The incision into the mediastinal pleura was extended as close as possible to the subclavian artery at the upper end and down to the pulmonary hilum, ensuring full exposure, with prompt application of electrocautery for hemostasis to minimize bleeding. To minimize the risk of damaging the PDA during ligation, we employed a technique where, after separating the upper and lower margins of the PDA with angled forceps, the PDA plane was freed from the outer posterior aspect of the descending aorta. We then inserted two 1 − 0 sutures, one above and one below the PDA margins, by passing them through the posterior aspect of the descending aorta. This approach simplified the procedure and minimized the risk of tissue damage during PDA ligation.

Our study had several limitations. It was a single-center retrospective study with a small sample size. This report reflects the early experience of our center and serves as an exploratory study. More objective and accurate conclusions will require studies with larger sample sizes in the future.

## Conclusion

During ECMO treatment for newborns with PPHN and large PDA, the direction of blood flow in the PDA should be closely monitored. PDA ligation is a feasible and reasonable intervention when pulmonary artery pressure decreases and a left-to-right shunt through the PDA becomes evident. However, performing PDA ligation during ECMO therapy still carries a high risk, necessitating close perioperative monitoring.

## Data Availability

The data that support the findings of this study are available on request from the corresponding author. The data are not publicly available due to privacy or ethical restrictions.

## Abbreviations

PDA: Patent Ductus Arteriosus
ECMO: Extracorporeal Membrane Oxygenation
PPHN: Persistent Pulmonary Hypertension
V-A: Venoarterial
PEEP: Positive End Expiratory Pressure
ACT: Activated Clotting Time
F: French
